# Truncation in the *tcdC *region of the *Clostridium difficile *PathLoc of clinical isolates does not predict increased biological activity of Toxin B or Toxin A

**DOI:** 10.1186/1471-2334-9-103

**Published:** 2009-06-28

**Authors:** Ruth Murray, Dave Boyd, Paul N Levett, Michael R Mulvey, Michelle J Alfa

**Affiliations:** 1 St. Boniface Research Centre, Winnipeg, MB, Canada; 2 Public Health Agency of Canada, Winnipeg, MB, Canada; 3 Public Health Agency of Canada, Saskatchewan, Canada; 4 Department of Medical Microbiology, University of Manitoba, Winnipeg, MB, Canada

## Abstract

**Background:**

The increased severity of disease associated with the NAP1 strain of *Clostridium difficile *has been attributed to mutations to the *tcdC *gene which codes for a negative regulator of toxin production. To assess the role of hyper-production of Toxins A and B in clinical isolates of *Clostridium difficile*, two NAP1-related and five NAP1 non-related strains were compared.

**Methods:**

Sequencing was performed on *tcdC*, *tcdR*, and *tcdE* to determine if there were differences that might account for hyper-production of Toxin A and Toxin B in NAP1-related strains. Biological activity of Toxin B was evaluated using the HFF cell CPE assay and Toxin A biological activity was assessed using the Caco-2 Trans-membrane resistance assay.

**Results:**

Our results confirm that Toxin A and Toxin B production in NAP1-related strains and ATCC 43255 occurs earlier in the exponential growth phase compared to most NAP1-nonrelated clinical isolates. Despite the hyper-production observed in ATCC 43255 it had no mutations in *tcdC*, *tcdR *or *tcdE*. Analysis of the other clinical isolates indicated that the kinetics and ultimate final concentration of Toxin A and B did not correlate with the presence or lack of alterations in *tcdC*, *tcdR *or *tcdE*.

**Conclusion:**

Our data do not support a direct role for alterations in the *tcdC *gene as a predictor of hyperproduction of Toxin A and B in NAP1-related strains.

## Background

Toxigenic *Clostridium difficile *causes *C. difficile*-associated diarrhea (CDAD) which is a predominant nosocomial infection in Canada as well as other countries [1-15]. In Manitoba, in 2008, there were 890 lab-confirmed cases of CDAD versus 535 total cases of infectious diarrhea due to all other bacterial enteric pathogens combined [16].

Recently published data indicates that although CDAD incidence varies by geographic region there is evidence that overall it is increasing [2,4,5,7,12-15,17]. In Quebec there were 35.6 cases per 100,000 population in 1991 versus 156.3 cases per 100,000 in 2003 [2]. The severity of infection as assessed by mortality rates has increased in certain geographic locations [2-4,7,8,15,18,19]. The *C. difficile *strain designated NAP1 (equivalent to PCR ribotype 027) was the most prevalent strain associated with the Quebec outbreak of CDAD [4,5,7,8,15] and it has been found in other parts of Canada as well [20]. The significant increase in incidence and disease severity reported for the Quebec outbreak have prompted investigations to determine if this strain has some unique virulence characteristics. This strain has the gene (*cdt*B) encoding binary toxin [4,18,21,22] and this has been suggested to contribute to disease severity [15,23,24]. The *tcd*C gene is the putative negative regulator for toxin production within the pathogenicity locus (PaLoc) of *C. difficile *[3,4,15,20,25] and deletions in this gene have been suggested to affect the regulatory function and account for the apparent high levels of toxin production of the NAP1 outbreak strain [4,20,25]. The initial studies used in vitro ELISA to quantify the production of Toxin A and B relative to the growth curve for the a collection of NAP1 strains that were toxinotype III compared to the a collection of clinical isolates that were toxinotype 0 [4]. Because there may be differences in the biological activity versus antigen detected, further data evaluating the biological levels of Toxins A and B are needed.

The objective of this study was to provide correlation between the kinetics of toxin production using functional assays and the growth kinetics of various clinical strains of *C. difficile*. The primary focus of this study was to evaluate the effects of the *tcdC *region deletions that have been described (18 bp and 1 bp deletions) on the kinetics of biologically active Toxin A and B production. This was done in the context of clinical isolates, thereby evaluating the role of this gene in the overall kinetics of toxin A and B production. Genetic analysis of the *tcdR *and *tcdE *regions was also performed to determine if deletions or mutations in these genes accounted for differences in toxin A and B production.

## Methods

### Bacterial strains

Eight strains of *C. difficile *were selected for analysis of the growth dynamics and toxin production. Three historical clinical isolates (strains1083, 81A330, and 79A292) were obtained from the culture collection at the National Microbiology Laboratory (NML). Two NAP1-related clinical isolates (strains 57A, 83) were provided by Dr. Paul Levett at the Saskatchewan Provincial Health Laboratory. Strains from the American Type Culture Collection included; ATCC 43255, ATCC 43594 (both are toxigenic strains) and ATCC 700057 (non-toxigenic strain).

### Polymerase chain reaction and sequencing

PCR was carried out with AmpliTaq Gold in PCR Buffer II (Applied Biosystems) with 0.2 mM dNTPs, 3 mM MgCl_2_, and various primer concentrations, at an annealing temperature of 58°C. The primers used in this study are listed in Table 1.

**Table 1:** Primers used in this evaluation.

Sequence	primer	Gene	Reference
AAAGAAGCTACTAAGGGTACAAA	tpi-F	*tpi*	[26]
CATAATATTGGGTCTATTCCTAC	tpi-R		[26]
AGATTCCTATATTTACATGACAATAT	tcdA-F	*tcdA*	[26]
GTATCAGGCATAAAGTAATATACTTT	tcdA-R		[26]
AATGCATTTTTGATAAACACATTG	tcdB-3	*tcdB*	this study
AAGTTTCTAACATCATTTCCAC	tcdB-4		this study
TCTCTACAGCTATCCCTGGT	PaL15	*tcdC*	[12]
AAAAATGAGGGTAACGAATTT	Pal16		[12]
TTTCATACATTTGTGCTGGG	cdd1-A	*tcdC*	this study
AATGCATTTTTGATAAACACATTG	tcdC-3		this study
TTCTAGATTTCATAAAAGATAC	TPR-1	*tcdR*	this study
CTGACATATTATGATATTCTTC	tcdB-UP		this study
GTTGTTTAGATTTAGATGAAAAGA	Lok6	*tcdE*	[33]
CTTGGTCTAATGCTATATGCGAG	PrimexA		[34]
CTTAATGCAAGTAAATACTGAG	cdtB-pos	*cdtB*	[24]
AACGGATCTCTTGCTTCAGTC	cdtB-rev		[24]

Strains were confirmed as *C. difficile *and putative toxigenic status determined by two PCR multiplexes, [26] one consisting of the tpi-F/R primers (*C. difficile *triose phosphate isomerase housekeeping gene) and the tcdA-F/R primers (toxin A), and one consisting of the PaL15/16 primers (*tcdC*), cdtB-pos/rev primers (binary toxin subunit B), and tcdB-3/4 primers (toxin B).

Genes and flanking regions were amplified for sequence analysis (Table 2) with primers TPR-1/tcdB-UP (*tcdR*), Lok6/PrimexA (*tcdE*), and cdd1-A/tcdC3 (*tcdC*). Oligonucleotides were synthesized, and dideoxy cycle sequencing carried out by the Genomics Core Facility of the NML.

**Table 2:** GeneBank Accession numbers for genes sequenced.

Strain	accession no. for gene region		
	*tcdR*	*tcdE*	*tcdC*
ATCC 43594	DQ912170	DQ902560	DQ870674
1083	DQ912171	DQ902562*	DQ272240**
79A292	DQ912172	DQ902561	DQ970676
81A330	DQ912173	n.d.^*a*^	DQ870675
57A	DQ912174	DQ902559	DQ272239
83	DQ912175	n.d.^*a*^	n.d.^*a*^

^*a *^n.d., not deposited as the sequence for this region was 100% identical to the same region from strain 57A.

* The 1 bp deletion is at position 272 of the DQ902562 gene sequence. The deletion causes a frameshift resulting in an in-frame stop codon at position 275–277 of the DQ902562 sequence.

** The 1 bp substitution that results in a transversion of C to A is at position 695 of the DQ272240 sequence.

Selective regions of the Toxin A and B were analyzed following [27] toxinotyping procedure using the B1C and B2N primers to amplify region B1 and A3C and A4N primers to amplify region A3.

Binary toxin PCR was performed following [28] procedure using the cdtB primers cdtBpos and cdtBrev.

### Growth conditions

*C. difficile *strains were grown on Tryptic soy agar containing 5% sheep blood (BA) (Oxoid Nepean, ON) that were incubated at 37°C in a Bactron anaerobic chamber (Sheldon Manufacturing Cornelius, OR). Prior to each experiment, 48 hour pure cultures on BA were subcultured into pre-reduced Brain Heart Infusion broth (BHI) (Difco Kanasas City, MO). After overnight growth at 37°C each culture was used to inoculate BHI (10 mls) or to provide a final bacterial concentration of ~10^3 ^cfu/ml. The inoculated BHI culture in Hungate tubes (screw cap with a rubber septum facilitated aspiration of samples) was incubated at 37°C and samples were taken at various time frames over 60 hours to determine viable count and toxin A and B titres using the methods described below.

### Bacterial Quantitation

Samples were serially diluted 1:10 in BHI and then 0.1 ml of each dilution was inoculated onto *Clostridium difficile *Moxalactam Norfloxacin (CDMN) agar plates (Oxoid Nepean, ON) using the spread plate technique. All counts were performed in triplicate after 48 hours incubation and the average ± standard deviation was determined.

Spores were detected using alcohol shock and quantitative count using the CDMN spread plate technique.

### Cell Culture

Human foreskin fibroblast (HFF) cells, were used for the Toxin B cytotoxin assay as described by Du et al. [30]. Cells were examined for cytopathic effect (CPE) at 48 hours and a monolayer with at least 50% cell rounding was considered positive for CPE [29]. The titer was reported as the reciprocal of the highest dilution positive for CPE. All results for Toxin B titres were reported as mean ± standard deviation from triplicate experiments. Purified Toxin B (Techlab Blackburg, VA), and myeloma media were included as positive and negative controls respectively. Bartel's (Carlsbad, CA) Toxin B control was also used as a positive control.

The Caco-2 cells (ATCC HTB 37), were maintained and used for the Toxin A tight-junction assay, in Transwell inserts (Corning Costar Corning, NY) as described by Du et al. [30] as modification of Grasset et al. [31] method. The transepithelial resistance (TER) was measured using Millicell-ERS (Millipore) and the monolayer was considered to be confluent when the TER was ≥ 400 Ω/cm^2 ^[30]. Samples were considered positive for Toxin A if a 50% drop in resistance was seen within 360 minutes of inoculating the insert. Purified Toxin A, Lot 0195005 (Techlab Blackburg, VA) at 460 ng/ml and cell culture media alone were included as positive and negative controls respectively.

## Results

**Figure 1:**
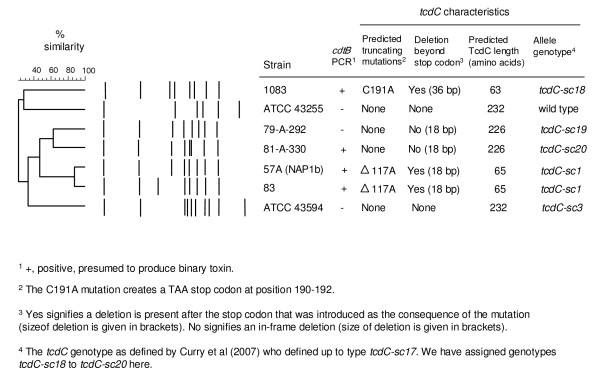
**Characteristics of *Clostridium difficile *strains**. All strains had *tcdA *and *tcdB *as the appropriate size of A3 and B1 amplicon was detected.[27] To assess NAP1 relatedness, PFGE analysis was performed using Sma1 as outlined by[32].

The objective of this study was to evaluate a variety (Figure 1) of clinical strains of *C. difficile *(NAP1 related and NAP1 non-related) to determine if there was a correlation between the genetic changes detected in the putative negative regulator gene (*tcdC*) and the growth kinetics and toxin production. The relatedness of strains was assessed by pulsed-field gel electrophoresis (Figure 1) using SmaI as the restriction enzyme following the method of [32].

The seven strains were assessed to confirm that both Toxin A and B genes were present and to ensure that there were not any large deletions within the B1 or A3 regions [27,33] of the pathogenicity locus (PaLoc). In addition sequencing was used to evaluate any changes in the positive regulator gene *tcdR*, the putative toxin secretory gene *tcdE*, as well as the putative negative regulator gene *tcdC*. The results of these analyses are shown in Figure 1.

**Figure 2:**
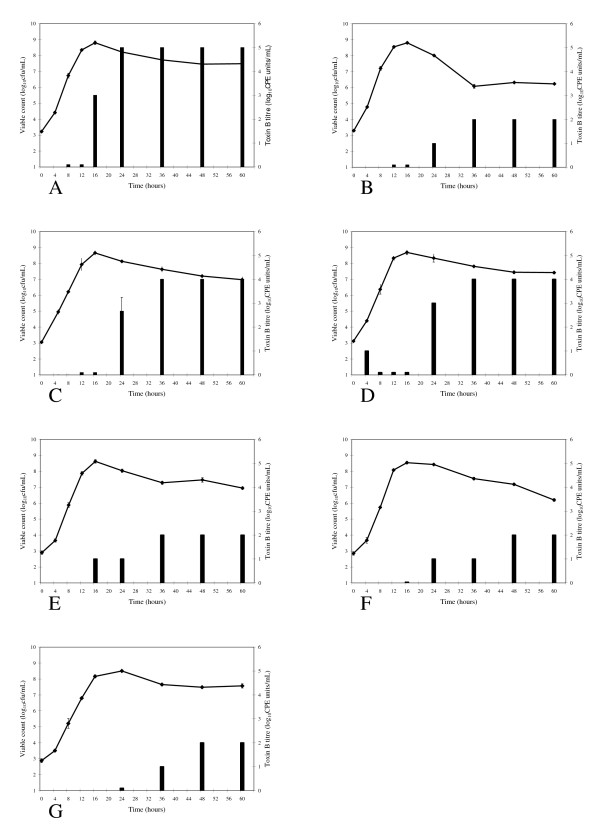
**Toxin B production over time for various strains of *C. difficile***. The viable count (line) and Toxin B production (black bars) were determined over 60 hours for isolates grown in BHI broth. The strains evaluated included; ATCC 43255 (A), ATCC 43594 (B), NAP1 clinical strain 57A (C), NAP1 clinical strain 83 (D), historical clinical strains 79A292 (E), 81A330 (F) and 1083 (G).

The results of the growth kinetic studies are shown in Figure 2. A clear segregation of strains was observed, as such strains were considered to be high-level producers (HLP) if the titre of Toxin B was ≥ 10^3 ^CPE units per ml by 48 hours incubation. The ATCC 43255 strain and the NAP1-related clinical isolates all produced high titres of biologically active Toxin B in broth culture whereas ATCC 43594 and the other three clinical isolates did not. The clinical strain 1083 was the slowest Toxin B producer (Figure 2G) and this was confirmed using multiple repeated growth curves (data not shown).

**Figure 3:**
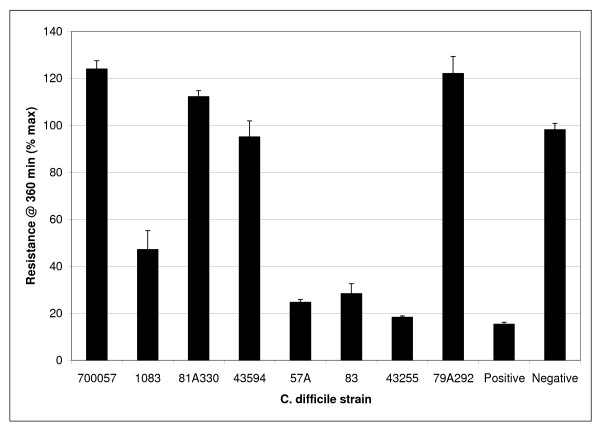
**Toxin A activity of *C. difficile *strains grown in BHI broth culture**. The Toxin A activity for 48 hour culture supernatants was determined using the trans-membrane resistance assay. The positive control consisted of purified Toxin A (460 ng/ml) and the negative control was BHI broth. Strain 700057 is a strain that in a non-toxin producing strain.

To determine if these strains had differing levels of biologically active Toxin A production, samples from cultures of each strain under the same growth conditions were evaluated at 24 and 48 hours post-inoculation. The ATCC 43255 strain produced more biologically active Toxin A than any of the other strains when 24 hour cultures were evaluated. By 48 hours (Figure 3), both NAP-related strains (83 and 57A) had reduced the trans-membrane resistance to < 30% whereas this TER was not seen for NAP1-unrelated strains.

**Figure 4:**
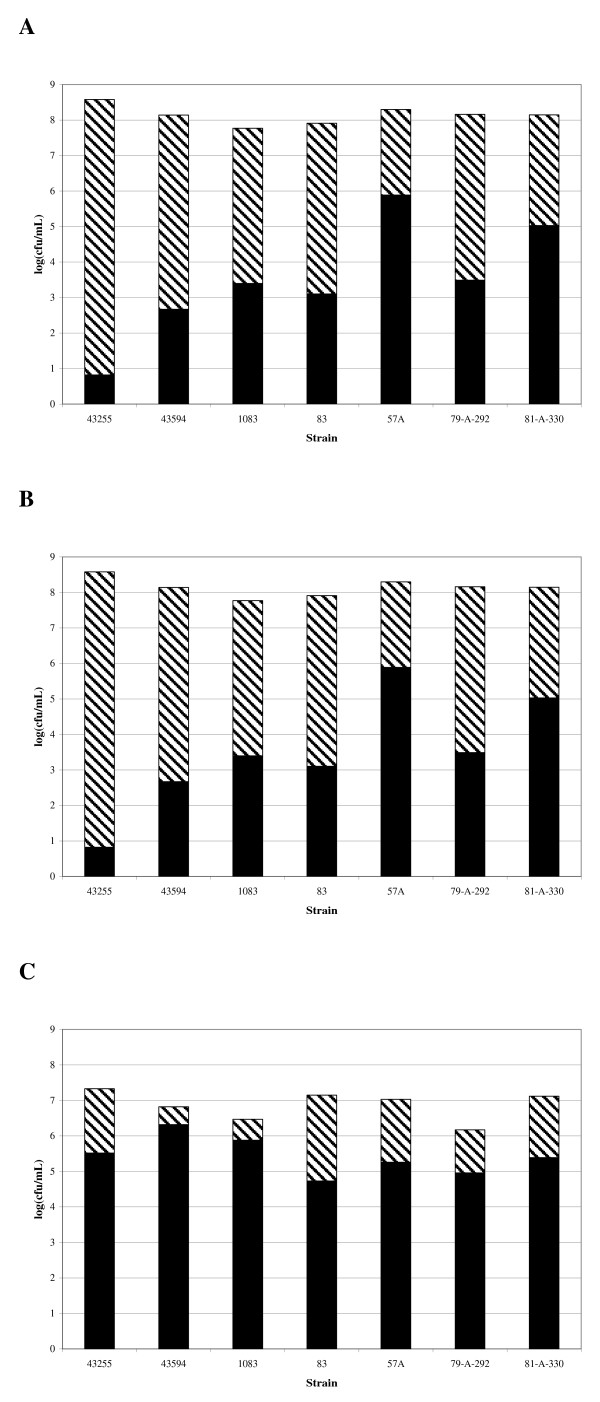
**Sporulation activity of *C. difficile *strains grown in BHI broth culture**. Spore production by strains of *C. difficile *grown in BHI broth for 24 (A), 48 (B) and 72 hours (C). The total viable count of *C. difficile *(□) was determined as well as an assessment using alcohol shock (as described in the Materials and Methods) to determine what portion was in the spore form (■). Each bar represents the average of triplicate testing. The 24 hour levels for strain 43255 were tested in triplicate on two separate occasions to verify that spore levels were consistently < 1 Log_10_.

Alcohol shock was used to determine what portion of the total viable count was in the spore form (Figure 4). The only major difference in spore formation noted was that strain 43255 had < 1 Log_10 _of spores after 24 hours incubation whereas all the other strains tested had between 10^2 ^to 10^5 ^spores/ml. By 48 and 72 hours incubation all strains had ~10^5 ^spores/ml from a total population of ~10^7 ^cfu/ml (i.e. viable count is predominantly in the vegetative form).

**Figure 5:**
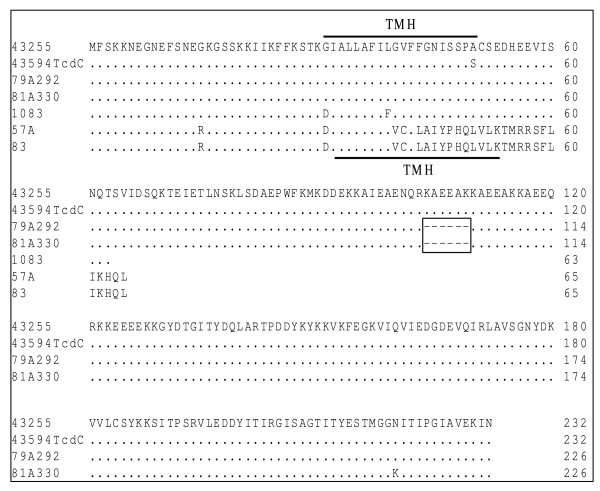
**Amino acid sequence alignment from *tcdC *gene of various strains of *C. difficile***. Amino acids identical to the ATCC 43225 sequence (shown) are indicated by periods, differences are shown, and the region of the six amino acid deletions (dashes) in strains 79A292 and 81A330 are boxed. The transmembrane helical regions (TMH) are indicated by a line.

The promoter region of *tcdC *(-35 to -10 bp upstream of the *tcdC *gene as described by Hunsdsburger et al. [34]) was sequenced to determine if the strains evaluated had any differences. An alignment of the sequences showed that all strains had identical promoter sequences (data not shown). Sequencing of the *tcdC *gene itself demonstrated that there were deletions that would lead to a premature termination codon in the protein sequence that would affect the expected length of the protein product (Figure 1). Alignment of the amino acid sequences is shown in Figure 5. It is apparent that for strains 1083, 57A and 83 that the proteins transcribed and translated from the *tcd*C gene would be substantially shorter compared to the other strains evaluated that had no deletions or premature stop codons. Although the truncated proteins are somewhat similar in length (63 vs 65 amino acids long), the position of the 1 bp change leads to very different amino acid sequence changes. Strain 1083 has a 1 bp substitution at position 191 that results in a C to A transversion that creates a premature stop codon and results in a 63 amino acid long protein that has an unchanged amino acid sequence up to position 174. Whereas the 1 bp deletion at position 117 in strains 57A and 83 results in a frame shift that leads to an altered amino acid sequence from position 117 onwards and a premature stop codon at positions 187–189 resulting in a predicted product of 65 amino acids.

The sequence analysis of the *tcdR *genes of the seven strains were identical (data not shown). Sequence analysis of *tcdE *revealed that only strain 1083 had any deletions. This 1 bp deletion in strain 1083 causes a frame-shift and leads to a stop codon after 18 amino acids compared to expected protein product of 166 amino acids for all other strains (Figure 1).

Because *cdtB *was detected by PCR, binary toxin was presumed to be produced by the NAP1-related strains 57A and 83 as well as 81A330 and 1083 which are NAP1-unrelated strains by PFGE (Figure 1).

## Discussion

Our results demonstrated that deletions in the *tcdC *gene cannot be used to predict hyperproduction of Toxin A or B in clinical isolates. Assessment of deletions in this gene could be very misleading if used as a diagnostic approach in patients with CDAD to try to predict who will have more severe disease. Furthermore, our data suggested that *tcdC *deletions may not be the sole factor responsible for the increased toxin production observed in the NAP-1 isolates. Studies of toxin production using isogenic mutants are warranted to confirm our results.

There are a number of possible explanations for what has been referred to as "hyperproduction" of toxins by some *C. difficile *strains and not by others. If the positive regulator *tcdR *was genetically altered in various strains it might result in differing toxin expression between strains. Our sequencing data indicates that the *tcdR *gene in all strains is identical ruling out genetic differences in the positive regulator gene as an explanation of differences in Toxin A and B production. An alternative mechanism for "hyperproduction" might be that the putative negative regulator *tcdC *is mutated thereby allowing earlier and more efficient expression of toxin due to the lack of a functional negative regulator. Although there were multiple changes found in *tcdC *in various strains of *C. difficile *(e.g. 57A, 83, and 1083) that resulted in predicted truncated proteins that are likely not active, these defects do not always correlate with increased toxin expression. Furthermore, strain ATCC 43255 does not have deletions or mutations in *tcdC *yet it has the highest levels of Toxin B and Toxin A production *in vitro *(higher even than the NAP1-related clinical isolates). Strains can differ in the proportion of the population that is in the spore form and this might explain the differences in secreted toxin levels. However, the growth conditions in our study did not result in variation in spore levels for the different strains evaluated. Therefore, the differences in toxin production observed cannot be explained by differences in spore form population. Our data therefore suggest that alterations in the *tcdC *gene sequence are not accurate predictors of hyper-production of Toxin B or Toxin A.

The ATCC 43255 strain is a known hyperproducer of Toxin B [6,10,36] and our data for biologically active toxin production in BHI broth culture support this determination. The use of ATCC 43255 as the "type strain" may be misleading as Warny et al. [4] assessed 25 Toxinotype 0 clinical isolates (non NAP1-related) and reported that hyperproduction of toxin is not common in non-NAP1 clinical isolates.

The TcdC protein has a predicted transmembrane domain [34] which was lost for the truncated product and altered amino acid sequence for strains 57A and 83. However, the transmembrane folding motif for TcdC was retained for strain 1083 despite the truncation of this protein. Despite this retained transmembrane motif for strain 1083 it is unlikely that the TcdC protein is fully functional when only a predicted 63 of the total 232 amino acids are present.

Another possible explanation for increased toxin activity in our experiments might be that secretion of the toxins outside the bacterial cell is increased in "hyperproducing" strains. Sequencing of *tcdE *indicates that there are no differences in this gene in the strains evaluated except for strain 1083 that has a 1 bp deletion that results in a truncated product of 18 amino acids compared to the expected size of 166 amino acids. If secretion was negatively impacted because the TcdE protein product was non-functional (i.e. unable to secrete toxins properly), this might explain why the levels of Toxin B were low in this strain despite the truncation to the *tcdC *gene that is suspected to predict toxin hyperproduction. Our study evaluated the levels of secreted toxin B, therefore, the levels of toxin detected in our assays would be low if the strain had decreased ability to secrete toxin. However, there are other strains (e.g. 79A292 and 81A330) that are low toxin producers that do not have defects in *tcdE*. Furthermore, neither ATCC 43255 nor the NAP1-related clinical isolates have defects in *tcdE *yet they produce high titres of biologically active Toxin B in broth culture.

Our data confirms previous reports [4,37] as near maximal biologically active Toxin B production was reached earlier in the growth curve (24 hours) of "hyperproducing" strains in BHI compared to "non-hyperproducing" strains (36 to 48 hours). We found that "hyperproducing" strains produced 100 to 1000 fold more Toxin B at 24 and 48 hours in BHI compared to "non-hyperproducing" strains. Our studies focused on biological activity of Toxin A and B whereas, Warny et al. [4] assessed total protein concentration irrespective of functionality, so this may explain the difference in ratios between "hyperproducing" and "non-hyperproducing" strains. Both studies assessed the culture supernatant from broth cultures as the source of secreted toxin. We used BHI broth and Warny et al. [4] used an Acambis proprietary broth media, so there may be some differences in the kinetics of toxin production due to different growth medium. In our studies *C. difficile *strain 43255 produced the highest levels of biologically active Toxin B in BHI broth culture compared to all other strains tested, yet it has no deletions in *tcdE*, *tcdC *or *tcdR*, therefore, our data do not support the conclusion by others [4,35] that the mutations found in *tcdC *of NAP1 strains accounts for their higher Toxin B production.

The recent study of Matamouros et al. [37] used cloning experiments and concluded that *tcdR*, which is a positive regulator for Toxin A and B production, was under the negative regulatory control of *tcdC*. They concluded that the TcdC protein product does not directly interact with the Toxin A promoter but rather interacts with TcdR to prevent it from functioning properly and thereby causing a negative impact on Toxin A production (i.e. it acts as a sigma-factor antagonist). They suggest that their data provide the first proof that *tcdC *is a negative regulator and furthermore, they suggest that epidemic strains have mutations in *tcdC *that result in hyper-production of toxin. Despite their elegant data to show how TcdR is negatively impacted by TcdC, their data do not provide an explanation for why the *C. difficile *ATCC strain 43255 is a hyper-producer of Toxins A and B (it has no *tcd*C mutations and has an intact *tcdR*). Furthermore, *C. difficile *strain 1083 has a predicted truncated TcdC yet it does not hyper-produce Toxin A or B. Whether the use of the glutamate dehydrogenase promoter for tcd*C *in these studies (rather than the actual tcdC promoter) has any role in the finding is unknown (all other genes cloned were under control of their natural promoter) [37].

The assays used in our study to assess biological activity are different so we cannot directly compare the titres of Toxin B to those of Toxin A. Despite this limitation our data does allow comparison of relative production of each toxin across a range of *C. difficile *strains. Strain 43255 produced significantly higher titres of biologically active Toxin B and Toxin A in broth culture compared to all other strains. Differences in the binding avidity, or kinetics of glucosyltransferase activity of Toxin A and B was not assessed. Previous studies have reported that mutation of tryptophan-101 in Toxin A (a similar effect was also seen for Toxin B) resulted in reduced glucosyltransferease activity [38]. Our results demonstrated that there was approximately 100–1000-fold higher titres of biologically active Toxin B production after 24 and 48 hours in broth culture for "hyper" versus "non-hyper" producing clinical isolates. Our results were obtained from the analysis of a small number of isolates that were chosen to represent sub-types of *C. difficile *that have been associated with CDAD. The control strains, ATCC 43255, 43594 and 700057, included the wild-type strain, a second toxigenic strain and a non-toxigenic strain respectively. It is not possible from our data or any of the currently published data to conclusively determine what gene(s) (if any) are responsible for "hyperproduction" of toxins in some strains of *C. difficile*. Furthermore, the significance of *in vitro *"hyperproduction" of Toxin A and B may or may not reflect what occurs *in vivo*. There have been no evaluations of humans infected with "hyperproducing" strains to establish that the titre of Toxin A or B/gram of stool is higher compared to levels in patients infected with non-hyper producing strains of *C. difficile*. However, the recent study by Freeman et al. [39] using the human gut model showed that post-exposure to clindamycin, the level of Toxin B production was similar for NAP1-related strain and a ribotype 001 strain.

Our data would suggest that the Caco2 transmembrane resistance assay is an insensitive test method for Toxin A as ≥ 460 ng/ml was needed to give a detectable trans-membrane electrical resistance drop whereas ≥ 150 ng/ml of Toxin B produced detectable CPE in HFF cells. Alternatively Toxin A activity may degrade more rapidly in BHI broth culture compared to Toxin B activity.

## Conclusion

In summary, our data do not support the role of mutations in *tcdC *as the sole basis for NAP1-related strains being hyper-producers of Toxins A and B. The production of isogenic *C. difficile *mutants has been described by [40] and this approach may be necessary to clarify the role of *tcdC*.

## Competing interests

The authors declare that they have no competing interests.

## Authors' contributions

All authors read and approved the final manuscript. MA contributed to the conceptual design, data analysis and writing. RM contributed to the conceptual design, data analysis and writing. PL contributed to the data analysis, writing, and experimental testing. DB contributed to the experimental testing (PCR & PFGE) and writing. MM contributed to the data analysis and writing.

## Pre-publication history

The pre-publication history for this paper can be accessed here:

http://www.biomedcentral.com/1471-2334/9/103/prepub
